# Immune Checkpoint Inhibitor (ICI) Induced Sinonasal Disease: Review of Literature and FDA Database

**DOI:** 10.1177/00034894241273192

**Published:** 2024-08-08

**Authors:** Kaitlynne Y. Pak, Wasiq Nadeem, Victor Lee, Dennis M. Tang, Arthur W. Wu

**Affiliations:** 1 Division of Otolaryngology, Head and Neck Surgery, Cedars-Sinai Medical Center, Los Angeles, CA, USA

**Keywords:** chronic rhinosinusitis, immune checkpoint inhibitors, sinonasal disorders and neoplasms

## Abstract

**Background:**

Immune checkpoint inhibitors (ICIs) are a rapidly expanding class of oncologic therapies whose mechanism of action can result in unique immune-related adverse events (irAEs) not seen in other cancer therapeutics. The objective of this study was to determine the presence of sinonasal irAEs with these medications.

**Methods:**

A case report of chronic rhinosinusitis with nasal polyps (CRSwNP) caused by an ICI is presented and was the impetus for this review. Review of the literature using Pubmed and Cochrane Database of Systematic Reviews was performed. Additionally, we searched the FDA adverse event reporting system (FAERS) database for sinonasal AEs in the 7 FDA-approved ICIs.

**Results:**

We demonstrate an emerging scientific literature describing cases of CRS associated with multiple ICIs with a particular predilection toward TH2 driven phenotypes. Review of the FAERS also demonstrates a small percentage of patients who report sinonasal complaints after initiating ICI therapy.

**Conclusion:**

Sinonasal symptoms and the development of CRS, in particular, are not currently well recognized as potential irAEs for ICIs. Increased awareness and further study may help to elucidate if these are more common than currently reported and if irAE-related CRS is a unique phenotype.

## Introduction

Immune checkpoint inhibitors (ICI) are a rapidly expanding class of oncologic therapies that involves antibodies targeting the cytotoxic T-lymphocyte-associated antigen-4 (CTLA-4), programmed cell death-1 (PD-1), and PD ligand 1 (PD-L1) pathways. Their utilization in cancer treatment has grown significantly in recent years, with ICIs being part of first-line treatment regimens for up to 22% of all cancers and 8% of head and neck cancers in 2020.^1^ With ICIs being considered in over a third of all cancer cases and utilized in nearly a quarter, it is essential to have a thorough understanding of both their benefits and risks.

Due to their mechanism of action, ICIs are responsible for unique immune-related adverse events (irAEs) not seen with other cytotoxic therapies. Guidelines exist for the management of irAEs involving 11 major organ systems, but there is a paucity of data and recommendations for sinonasal irAEs.^2^ We aim to report a case of severe chronic rhinosinusitis with nasal polyps (CRSwNP) after ICI initiation, review current literature, and analyze the FDA adverse event database for sinonasal irAEs with ICI therapy. In doing so, we intend to uncover the prevalence of sinonasal irAEs, detail specific irAEs for which clinicians must monitor, and provide guidance on their management.

## Methods

We summarize a single case involving severe onset of CRSwNP with pembrolizumab which was the impetus for this review. A preliminary search of PubMed and the Cochrane Database of Systematic Reviews was conducted, and no current systematic reviews or scoping reviews were identified on the topic of sinonasal disease associated with the following FDA-approved ICIs: CTLA-4 inhibitors (ipilimumab), PD-1 inhibitors (cemiplimab, nivolumab, and pembrolizumab), and PD-L1 inhibitors (atezolizumab, avelumab, and durvalumab). This literature review was conducted against chronic rhinosinusitis with and without nasal polyps (CRSwNP and CRSsNP), eosinophilic chronic rhinosinusitis (ECRS), and allergic fungal rhinosinusitis (AFRS). Because of the relatively recent adoption of these medications and the lack of studies, a systematic review was not performed. We also performed a data analysis of the 7 FDA-approved ICIs for sinonasal complaints reported in the FDA adverse event reporting system (FAERS) database. Data was collected from Jan 1, 2019 to June 30, 2023. Any events attributed to a sinonasal origin were included, with concurrent analysis performed using Microsoft Excel.

## Results

We report a 43-year-old female who developed new and progressive nasal obstruction, rhinorrhea, post-nasal drip, and anosmia after initiating pembrolizumab therapy for endometrial cancer. On exam, the patient had bilateral turbinate hypertrophy and large nasal polyps. The patient also experienced an increase in blood eosinophil count over the course of pembrolizumab treatment; eosinophil count was 160 cell/µL at the start date, 480 at 4 months, and 1110 at 7 months of treatment. The patient was placed on intranasal steroid spray but has deferred surgical treatment due to her concomitant oncologic treatment. This presentation and course prompted the following literature and database review.

Literature review within the CTLA-4 inhibitors yielded 1 associated case of ECRS and 1 case of CRS. Of the PD-1 inhibitors, there was 1 case of ECRS, 7 cases of CRSwNP including our study, 1 case of AFRS, and 13 cases of CRSsNP. No associated cases were identified in association with PD-L1 inhibitors (Table 1).

**Table 1.** Literature Review of Sinonasal irAEs With ICI Therapy.

ICI Class	Drug	Literature
CTLA-4	Ipilimumab	Watanabe et al,^3^ (Respirol Case Rep)—ECRS Dein et al,^4^ (Immunother)—CRS
PD-1	Cemiplimab	N/A
	Nivolumab	Watanabe et al,^3^ (Respirol Case Rep)—ECRS Dein et al,^4^ (Immunother)—CRS Rembalski et al^5^ (J Immunother)—CRSwNP Kassem et al^6^ (Asian Pac J Allergy Immunol)—RSwNP
	Pembrolizumab	Hintze et al,^7^ (J Rhinol)—CRSwNP Tzoumpa et al^8^—CRSwNP Standiford et al^9^ (Int Forum Allergy Rhinol)—CRS Krane et al,^10^ (Annals)—AFRS Pak et al—CRSwNP
PD-L1	Atezolizumab	N/A
	Avelumab	N/A
	Durvalumab	N/A

Abbreviations: AFRS, allergic fungal rhinosinusitis; CRS, chronic rhinosinusitis not otherwise specified; CRSwNP, chronic rhinosinusitis with nasal polyps; CTLA-4, cytotoxic T-lymphocyte-associated antigen-4; PD-1, programmed cell death-1; PD-L1, PD ligand 1; ECRS: eosinophilic chronic rhinosinusitis

Upon further review of the FAERS database, 133,118 irAEs were reported for FDA-approved ICIs. The overall numberi of sinonasal irAEs was 0.62% with 826 cases, including 405 cases of epistaxis, 142 rhinorrhea/rhinitis, 127 sinonasal congestion or sneezing, 35 nonspecific sinonasal disease, 31 allergic rhinitis, 11 CRSwNP, and 75 other nonspecific sinonasal complaints. The greatest number of sinonasal irAEs was reported for patients taking nivolumab (282) and the highest number of sinonasal irAEs was reported for patients taking atezolizumab (0.94%; Table 2).

**Table 2.** FAERS Database Review of Sinonasal irAEs.

Mechanism	Drug	Total FDA adverse events (#)	Sinus related FDA adverse events (#; 2019-2023)	Percentage (% total)
CTLA-4	Ipilimumab	2023: 2584 2022: 4534 2021: 3934 2020: 3572 2019: 3706 Total: 18 330	Epistaxis: 28 Rhinorrhea: 16 Nasal congestion: 10 Sneezing: 8 Allergic rhinitis: 4 Sinus disorder: 2 Nasal dryness: 2 Nasal polyps: 2 Paranasal sinus hypersecretion: 1 Sinus polyp: 1 Sinonasal obstruction: 2 Total: 76	0.41%
PD-1	Cemiplimab	2023: 93 2022: 147 2021: 138 2020: 138 2019: 134 Total: 650	Epistaxis: 2 Sneezing: 1 Total: 3	0.46%
	Nivolumab	2023: 5499 2022: 10 258 2021: 9731 2020: 9703 2019: 10 531 Total: 45 722	Epistaxis: 101 Rhinorrhea: 63 Nasal congestion: 23 Sneezing: 17 Nasal dryness: 13 Sinus disorder: 8 AR: 8 Rhinalgia: 8 Nasal ulcer: 7 Paranasal sinus HS: 4 Nasal polyps: 4 Nasal septum perf: 4 Sinus congestion: 3 Nasal discomfort: 3 Nasal disorder: 2 Sinonasal obstruction: 4 Nasal inflammation: 2 Nasal crusting: 2 Allergic sinusitis: 1 Sinus polyp: 1 Nasal turb hypertrophy: 1 VM rhinitis: 1 CRSwNP: 1 CRS (eosinophilic): 1 Total: 282	0.62%
	Pembrolizumab	2023: 8156 2022: 10 659 2021: 7574 2020: 6190 2019: 5803 Total: 38 382	Epistaxis: 118 Rhinorrhea: 46 NC: 32 Sinus disorder: 11 Sinus congestion: 8 Sneezing: 6 AR: 6 Nasal dryness: 6 Rhinalgia: 4 Paranasal sinus hypersecretion: 5 Nasal polyps: 2 Allergic sinusitis: 1 Nasal inflammation: 1 Sinus inflammation: 1 Sinus hemorrhage: 1 CRS(eosino-): 1 Total: 249	0.65%
PD-L1	Atezolizumab	2023: 4430 2022: 5861 2021: 4627 2020: 3103 2019: 2330 Total: 20 351	Epistaxis: 141 NC: 11 Rhinorrhea: 10 Nasal dryness: 8 Sinus disorder: 6 AR: 6 Sneezing: 2 Nasal cyst: 2 Rhinalgia: 2 Nasal disorder: 1 NS perforation: 1 Nasal necrosis: 1 Nasal ERD: 1 Total: 192	0.94%
	Avelumab	2023: 290 2022: 582 2021: 509 2020: 471 2019: 439 Total: 2237	Epistaxis: 5 Total: 5	0.22%
	Durvalumab	2023: 1164 2022: 1562 2021: 1718 2020: 1559 2019: 1443 Total: 7446	Epistaxis: 9 Sinus congestion: 4 Sneezing: 2 Rhinorrhea: 1 Sinus disorder: 1 Sinus pain: 2 Total: 19	0.26%

## Discussion

The proposed mechanism behind irAEs revolves around T-cell activation, infiltration, and local inflammation. There are few reports in the literature describing sinonasal disease in the setting of ICI therapy.^3-10^ Our data suggests that ICIs may both aggravate and initiate sinonasal disease, particularly CRS. Unfortunately, specific phenotype/endotype was only noted in some studies, but there seems to be a preponderance of TH2-mediated CRS endotypes. Sinonasal irAEs, and specifically CRS, were also found in the FAERS database. While epistaxis was most common, several reported categories of sinonasal irAEs may reflect undiagnosed CRS: rhinorrhea/rhinitis, sinonasal congestion or sneezing, nonspecific sinonasal disease, allergic rhinitis, and nonspecific sinonasal complaints. While ICIs are typically employed to treat life-threatening malignancies and the reported numbers of sinonasal irAEs appears relatively low, clinicians should be aware of the potential to develop CRS, a chronic condition known to significantly decrease long-term overall quality of life. Additionally, because sinonasal symptoms may be considered normal and may fluctuate for patients with comorbid allergy, for example, these irAEs may be under-reported. To our knowledge, this is the first literature review and concurrent FAERS database review of sinonasal irAEs. Patients undergoing ICI therapy should be monitored for new or exacerbated sinonasal disease, especially CRSwNP. These patients may be managed medically and surgically, allowing them to continue their cancer treatment. Future prospective and comparative studies would be helpful to better understand the prevalence of sinonasal irAEs, whether irAE-related CRS is a unique phenotype/endotype, the response to standard medical and surgical treatment, and whether eventual stoppage of the therapy provides resolution of sinus inflammation and symptoms.

## Conclusion

ICI therapy, which involves CTLA-1, PD-1, and PD-L1 antibody blockade, is an increasingly popular oncologic treatment with unique irAEs. Sinonasal symptoms and the development of CRS, in particular, are not currently well recognized as potential irAEs for ICIs. Increased awareness and further study may help to elucidate if these are more common than currently reported and if irAE-related CRS is a unique phenotype.
